# Aggressive and malignant pituitary tumours: does the sex matter?

**DOI:** 10.1007/s11102-026-01656-y

**Published:** 2026-03-07

**Authors:** Anna Lucia Carretti, Chrysi Kaparounaki, Gérald Raverot

**Affiliations:** 1 Department of Experimental Medicine, Sapienza University of Rome, Rome, Italy; 2 Endocrinology Department, Reference Center for Rare Pituitary Diseases HYPO, “Groupement Hospitalier Est” Hospices Civils de Lyon, Bron, France; 3 Claude Bernard Lyon 1 University, Villeurbanne, France; 4 National and Kapodistrian University of Athens, Athens, Greece; 5 Cancer Research Center of Lyon, Inserm U1052, CNRS UMR5286, Lyon, France

**Keywords:** Aggressive pituitary tumours (APT), Pituitary carcinoma (PC), Cushing’s disease, Lactotroph pituitary tumours, Corticotroph pituitary tumours, Sex-related differences

## Abstract

**Purpose:**

Aggressive pituitary tumours (APT) and pituitary carcinomas (PC) are rare pituitary neoplasms and occur more frequently in men. Corticotroph and lactotroph tumours represent the most frequent secreting subtypes, showing different sex-related distribution, clinical features, and behaviour compared with benign disease. This review evaluates whether these sex-related differences persist in APT/PC and their clinical and therapeutic implications.

**Methods:**

A literature review of case reports, series, and clinical studies, published between 2000 and October 2025, reporting sex-related data on corticotroph and lactotroph APT/PC, was conducted. Clinical presentation, pathological features, and outcomes were extracted and analysed by sex.

**Results:**

93 corticotroph APT/PC (38 W, 55 M) and 80 lactotroph APT/PC (29 W, 51 M) were included. Among corticotroph APT/PC, men more commonly had non-functioning lesions (36.5%, vs. 17.6%), showed loss of hormonal secretion (7.7%, *n* = 4/52), and had invasive disease (100%, vs. 73.3%, p: 0.03). Women more frequently harboured Crooke’s cell tumours (26.3% vs. 5.5%, p: 0.006) and showed complete radiological responses (22.2%, vs. 9.1%). In lactotroph APT/PC, men had giant tumours (27.3%, vs. 5.0%), invasive disease (83.3%, vs. 63.6%), higher prolactin levels, and dopamine agonist resistance. Complete tumour responses to temozolomide occurred exclusively in women (22.7%, *n* = 5/22, p: 0.02), whereas partial durable responses predominated in men (46.2%, *n* = 18/39).

**Conclusion:**

Male sex is associated with higher prevalence and more aggressive disease, whereas women achieve more profound tumour and hormonal responses. Sex-specific stratification may support personalized management of these rare pituitary tumours.

**Supplementary Information:**

The online version contains supplementary material available at 10.1007/s11102-026-01656-y.

## Introduction

The latest European clinical practice guidelines [1] define aggressive pituitary tumours (APT) as invasive lesions with unusually rapid tumour progression, within 6 months, or clinically relevant tumour progression despite optimal medical treatments and/or surgery and radiation therapy. Pituitary carcinomas (PC), on the other hand, are diagnosed based on the presence of metastases. APT/PC have a combined estimated prevalence of 0.5–2% [2], with PC accounting for less than 0.1% of all clinically relevant pituitary lesions [2].

Data from the two European surveys [3, 4] show a higher prevalence of APT/PC in men accounting for nearly 65% of cases. Corticotroph (corticoPiT) and lactotroph tumours (prolactinomas or lactoPiT) were the most common subtypes, with frequencies of 30–45% and 24–32%, respectively. Men represented 60% of APT/PC patients with Cushing’s Disease (CD) [4], and nearly 70% of those with prolactinomas. In a recent systematic review and metanalysis on PC, Raymond et al. [5] reported an equal sex distribution for corticoPiT, and a men predominance in lactoPiT (62.7% vs. 37.3%). This contrasts with the higher female prevalence observed in cohorts of non-aggressive and non-malignant pituitary tumours for both subtypes.

As a matter of fact, Cushing’s Disease and corticoPiT show a women-to-men ratio of 3–8:1 [6–8]. However, in men, CD appears to occur at a younger age than in women [7], to be more clinically severe, and to be associated with a higher prevalence of hypokalaemia and osteoporosis complicated by vertebral fractures [6, 8]. Moreover, from a molecular point of view, these tumours in men are enriched for genes involved in tumour development [9] and less frequently harbour somatic *USP8* mutations, which are linked to postoperative remission but higher risk of recurrence [10].

Lactotroph tumours show a women-to-men ratio of approximately 10:1 [11, 12], but this difference is age-related and disappears after menopause [13]. They most frequently occur as microprolactinomas (maximal diameter, dmax <1 cm), which are responsible for menstrual irregularities in women of reproductive age. In contrast, in men, they typically present at an older age as macrotumours (dmax ≥1 cm), causing symptoms related to mass effect. These tumours are also often invasive and display a higher Ki-67 index [14, 15], both features associated with aggressive behaviour [3, 4]. Male sex, particularly when associated with cavernous sinus invasion, is also associated to resistance to dopamine agonist (DA) treatment [16]. LactoPiT in men are characterised by lower expression of Estrogen Receptor α (ERα) [17], which is related to higher tumour grade, resistance to treatment and overall worse prognosis. Other genes seem to be more frequently mutated in men, such as *SF3B1* (Splicing Factor 3 b 1), linked to DA resistance and poorer prognosis [18, 19].

First line treatment for APT/PC is temozolomide (TMZ), an alkylating agent which has showed efficacy in about two-thirds of patients [1]. In case of resistance or progression after TMZ, European guidelines suggest immune checkpoints inhibitors (ICIs) [1]. Other options, such as anti-VEGF monoclonal antibody (bevacizumab), tyrosine kinase inhibitors (TKI), mTOR inhibitors, and peptide receptor radionuclide therapy (PRRT), have been reported [20] with variable results.

The aim of this Pituitary special issue is to assess the gender gap in pituitary diseases. Studies specifically addressing sex-related differences in aggressive and malignant pituitary tumours remain scarce. In this review, we focus on the relevance of sex in APT/PC, with particular emphasis on corticotroph and lactotroph subtypes. These tumours are not only the most frequently reported in APT/PC series but also show distinct sex-specific features in benign and non-aggressive cohorts.

## Methods

In this narrative review, we included articles published from 2000 onwards, comprising case reports, case series and clinical studies both on aggressive and metastatic corticoPiT and lactoPiT, functioning and non-functioning (NF) histologically confirmed, provided that sex-disaggregated data were available. Relevant reviews, meta-analyses and the reference lists of included studies were also screened. Exclusion criteria included articles published before 2000, studies not reporting on aggressive or metastatic disease, studies lacking sex-specific data, and non-English-language publications.

Data on clinical presentation, treatment and histopathology were extracted and analysed according to sex.

Tumour invasion was defined as tumour extension into the cavernous sinus and/or the sphenoid sinus assessed at magnetic resonance imaging. Information was retrieved either from the text of the included articles, or from images of magnetic resonance imaging (MRI) when available, and defined by Knosp grade ≥ 3 for cavernous sinus [21].

Tumour and hormonal responses to treatment were assessed, when feasible, according to the RECIST criteria for solid tumours [22] and are reported in the present study as the best response achieved.

### Statistical analysis

Data distribution was considered non-normal. Continuous variables are expressed as medians with interquartile ranges (IQR), while dichotomous and ordinal variables are expressed as frequencies and percentages. Comparisons between sexes for continuous variables were performed using the Mann-Whitney U test for unpaired data, and differences in dichotomous variables were assessed by Fisher’s exact test and ꭓ^2^ test where needed.

Statistical significance was defined as a p-value < 0.05 and is reported in the text only when significant. All statistical analyses and graphical illustrations were conducted using GraphPad Prism 8 and R 4.3.3.

## Results

### Corticotroph APT/PC

Among a total of 93 cases of APT/PC corticoPiT (47.3% APT and 48.4% PC, 4.3% either APT or PC), 59.1% (*n* = 55) were men (M) [4, 20, 23–52] and 40.9% (*n* = 38) women (W) [4, 27, 28, 34–38, 42, 48, 52–70] (Supplementary Table 1). Relevant clinical and pathological data are summarised in Table 1.

**Table 1:** Clinical and pathological features in corticoPiT according to sex

Clinical features	Total	Men	Women	*p*
Number of cases	93	55 (59.1%)	38 (40.9%)	N.A.
APT, *n*	44	26 (59.1%)	18 (40.9%)	N.A.
PC, *n*	45	28 (62.2%)	17 (37.8%)	N.A.
Undetermined APT/PC, *n*	4	1 (25.0%)	3 (75.0%)	N.A.
Age at diagnosis (*n* = 88/51/37)	46 [39.8–54.3]	47 [38.5–54.5]	45 [40.0–53.0]	0.8
Age at metastases (*n* = 22/12/10)	54.5 [48.0–60.5]	56 [50.0–64.8]	53.5 [43.3–59.0]	0.4
Metastases (*n* = 42/27/15)	Bone 19 (40.2%) Liver 20 (47.6%) Meningeal 9 (33.3%) Intracranial/brain 11 (40.1%) Other 6 (14.3%)	Bone 12 (44.4%) Liver 9 (33.3%) Meningeal 7 (25.9%) Intracranial/brain 6 (22.2%) Other 3 (11.1%)	Bone 7 (46.7%) Liver 11 (73.3%) Meningeal 2 (13.3%) Intracranial/brain 5 (33.3%) Other 3 (20.0%)	N.A.
Functioning at diagnosis	61/86 (70.9%)	33/52 (63.5%)	28/34 (82.4%)	0.09
NF to F during follow-up	14/86 16.3%	9/52 17.3%	5/34 14.7%	> 0.9
F to NF during follow-up	4/86 (4.6%)	4/52 (7.7%)	0/34 (0%)	0.15
Macrotumour at diagnosis	37/43 (86.1%)	21/22 (95.5%)	16/21 (76.2%)	0.09
Invasion at diagnosis	29/33 (87.9%)	18/18 (100%)	11/15 (73.3%)	0.03
Invasion (after)	40/42 (95.2%)	22/22 (100%)	18/20 (90.0%)	0.22
First line treatment	Surgery 43/46 (93.5%) steroidogenesis inhibitors 2/46 (4.3%) CHT 1/46 (2.1%)	Surgery 24/25 (96%) Steroidogenesis inhibitors 1/25 (4%)	Surgery 19/21 (90.5%) steroidogenesis inhibitors 1/21 (4.8%) CHT 1/21 (4.8%)	N.A.
Pituitary surgeries (median number)	2 [2.0–3.0]	3 [2.0–3.0]	2 [1.0–3.0]	0.1
Radiotherapy	83/89 (93.3%)	51/54 (94.4%)	32/35 (91.4%)	0.7
Adrenal steroidogenesis inhibitors	24/88 (27.3%)	13/52 (25.0%)	11/36 (30.5%)	0.63
Pasireotide	24/88 (27.3%)	12/52 (23.1%)	12/36 (33.3%)	0.33
Bilateral adrenalectomy	34/71 (47.9%)	19/41 (46.3%)	15/30 (50.0%)	0.8
Tumour progression after BADX	19/34 (55.9%)	9/19 (47.4%)	10/15 (66.7%)	0.3
Follow-up duration (*n* = 40/25/15)	9.8 [5.6–14.3]	11 [7.0–19.0]	9 [5.3–10.0]	0.2
Deaths	16/73 (21.9%)	9/41 (21.9%)	7/32 (21.9%)	> 0.99
Time to death from diagnosis	9.9 [6.0–14.0]	9.5 [7.8–13.3]	8.8 [5.3–14.0]	0.97
Pathology				
Crooke cell tumours	13/93 (14.0%)	3/55 (5.5%)	10/38 (26.3%)	0.006
Ki67 ≥ 3% (general)	44/64 (68.8%)	27/38 (71.1%)	17/26 (65.4%)	0.78
Ki67 ≥ 10% (general)	20/64 (31.3%)	13/38 (34.2%)	7/26 (26.9%)	0.6
Ki67 ≥ 3% (before)	14/20 (70.0%)	6/10 (60.0%)	8/10 (80.0%)	0.63
Ki67 ≥ 10% (before)	4/20 (20.0%)	2/10 (20.0%)	2/10 (20.0%)	> 0.99
Ki67 ≥ 3% (after)	12/14 (66.7%)	7/9 (77.8%)	5/5 (100%)	0.5
Ki67 ≥ 10% (after)	7/14 (50.0%)	5/9 (55.6%)	2/5 (40.0%)	> 0.99
Proliferative (general)	26/30 (86.7%)	17/19 (89.5%)	9/11 (81.8%)	0.91
APT/PC specific treatments				
Temozolomide	85/88 (96.6%)	52/52 (100%)	33/36 (91.7%)	0.06
ICI	17/88 (19.3%)	11/52 (21.1%)	6/36 (16.7%)	0.78
BVZ	14/88 (15.9%)	10/52 (19.2%)	4/36 (11.1%)	0.38
CHT	5/88 (5.7%)	3/52 (5.8%)	2/36 (5.5%)	> 0.99
Everolimus	4/88 (4.5%)	2/52 (3.8%)	2/36 (5.5%)	> 0.99
PRRT	2/88 (2.3%)	0/52 (0%)	2/36 (5.5%)	0.16

Continuous variables are expressed as median with interquartile range; age, follow-up duration and time to death are expressed in years. Dichotomous and ordinal variables are expressed as frequencies and percentages. Comparisons between sexes for continuous variables were performed using the Mann-Whitney U test for unpaired data, and differences in dichotomous variables were assessed by Fisher’s exact test or ꭓ^2^ test whenever appropriated. Statistical significance was defined as a p-value < 0.05

*APT* aggressive pituitary tumour, *BADX* bilateral adrenalectomy, *BVZ* bevacizumab, *CHT* chemotherapy, *F* functioning, *ICI* immune checkpoint inhibitors, *NA* not available, *NF* non-functioning, *PC* Pituitary Carcinoma, *PRRT* peptide receptor radionuclide therapy

“Before” and “after” refer, respectively, to the period before and after the evidence of aggressive/malignant behaviour

A male predominance was observed in both APT and PC. Median follow-up from diagnosis was similar both sexes (Table 1). Age at diagnosis was comparable between sexes, with a median of 47 years (IQR 38.5–54.5) in men and 45 years (IQR 40.0–53.0) in women. Metastases occurred at a median age of 56 years (IQR 50.0–64.4) in men and slightly earlier in women, without statistical significance. In women the most common metastases localisation was the liver, whereas in men, they more frequently involved bones.

Functioning (F) tumours at diagnosis were 82.4% in women and 63.5% in men (Table 1); whereas non-functioning (NF), or silent, corticoPiT at diagnosis were observed mainly in men, accounting for 36.5% of cases (*n* = 19/52), compared with 17.6% in women (*n* = 6/34). All the NF cases had a histological diagnosis of corticoPiT. A shift from NF to F was observed in both sexes: 5 cases in women and 9 cases in men. Conversely, loss of secretory activity occurred exclusively in men (Table 1). Functioning status changed before evidence of overt aggressiveness in 4 men and one woman: from NF to F in 3 patients (1 W and 2 M), and from F to NF in 2 men. In 6 cases (4 M and 2 W) this shift happened either when the tumour was already recognised as aggressive or at the same time of evidence of aggressiveness: 3 men and 2 women shifted from NF to F, and one man from F to NF. For the remaining patients timing could not be established.

Data on tumour volume at diagnosis was available only for 40.0% of men and 55.3% of women. In both sexes, the majority were macrotumours (dmax ≥ 1 cm), identified in 95.5% of M and 76.2% of W (Table 1); giant tumours (dmax ≥ 4 cm) were rare: reported in 1/22 man and 2/21 women. Interestingly, microtumours (dmax < 1 cm) at initial presentation were observed exclusively in women, representing 14.3% (*n* = 3/21, 2 PC and 1 APT) of cases with available information. In one case [58], severe treatment-resistant Cushing’s disease, progressed to overt tumour growth and metastases following bilateral adrenalectomy. In another patient [57] rapid progression occurred in a tumour with a high proliferative index (Ki-67 = 40%) after initial surgery and severe hypercortisolism at initial presentation. In the third case [55], tumour recurrence after initial surgery showed a transition from densely to sparsely granulated corticoPiT, which is associated with aggressiveness [71]. These observations indicate that, especially in women where microadenomas are more common, even small lesions may undergo aggressive or malignant evolution, highlighting the need for careful follow-up.

Data on radiological tumour invasion at clinical presentation or prior to any treatment are limited. Among cases with available information, invasion was reported in 73.3% of women and in 100% of men, a difference that was statistically significant (p: 0.03) (Table 1). In 10 cases (4 M and 6 W), invasion developed when aggressive behaviour was more evident: 2 of them were not invasive at first presentation, and for the remaining 8 this information was not available. In total, 90.0% of women and 100% of men had invasive tumour at initial diagnosis and after evidence of aggressiveness (Table 1).

First line treatment was pituitary surgery for the vast majority of women and men; 2 patients (1 M and 1 W) were treated with steroidogenesis inhibitors first, and one woman received chemotherapy due to a misdiagnosis (Table 1). All patients underwent at least one pituitary surgery, except for one woman who had a biopsy followed by other lines of treatment [38], and 4 patients (3 women and 1 man) for whom surgical information was missing [52]. Postoperative radiotherapy was administered in 93.3% of patients (Table 1), and it always preceded the evidence of aggressive behaviour, except for 3 tumours which were diagnosed as APT/PC at diagnosis [24, 47, 48, 62]. Conventional medical treatment with adrenal steroidogenesis inhibitors and/or pasireotide was administered in about one third of patients, without significant differences between sexes (Table 1).

Bilateral adrenalectomy (BADX) to control refractory hypercortisolism was performed in similar proportion of men (46.3%) and women (50.0%) (Table 1). In 14 patients (8 W and 6 M) it was performed before the onset of overt aggressiveness, while in 11 (5 W and 6 M) it was performed after the diagnosis of aggressive/malignant behaviour. This information was not available for the remaining 9 patients (2 W and 7 M). Corticotroph tumour progression after BADX, formerly known as Nelson’s syndrome, was observed in 66.7% of women and 47.4% of men without statistical significance (Table 1). In 4 cases (2 M and 2 W) tumour progression occurred in an already defined aggressive disease (after TMZ in 2 cases), while in 11 (4 M and 7 W) it happened before or at the time of evidence of aggressive behaviour.

No patient received adjuvant treatment after BADX. However, in one man [33] neoadjuvant RT was performed before BADX while he was undergoing TMZ treatment and it possibly prevented further disease progression. Additionally, one woman without previous BADX showed tumour progression [60], likely due to mitotane-induced adrenolysis.

Pathological information was limited. In corticoPiT, the Crooke’s cell subtype, diagnosed on the presence of hyaline changes, is considered aggressive [71]. It was significantly more frequent in women than in men (p: 0.006) (Table 1), and all information was retrieved by samples before the evidence of aggressive behaviour.

Ki-67 index, a proliferative marker associated with tumour behaviour in APT/PC cohorts [2, 3], was assessed on the primary tumour in 68.8% (*n* = 64) of patients. For 3 patients (2 men and 1 woman) only categorical information (low vs. high) was reported. At the first available surgery, values of Ki-67 ≥ 3% and Ki-67 ≥ 10% were observed in similar proportion of men and women, with no significant difference (Table 1).

For 29/64 (17 M and 12 W) patients with available Ki-67 it was possible to assess Ki-67 values before and/or after the evidence of aggressive or malignant behaviour. Detailed information can be found in Table 1. No relevant differences were found between sexes both at initial presentation and after evidence of aggressiveness. It was possible to evaluate the Ki-67 trend for only 8 patients: in 5 (2 W and 3 M) it increased in subsequent surgeries and reached levels higher than 10% in all, while it decreased in 3 (2 W and 1 M) to levels of 5 and 1%. These data suggest that Crooke cell tumours, considered a morphologic feature of aggressiveness, are more frequent in women, while Ki-67 index was not significantly different between sexes. The other markers of aggressiveness (p53 and number of mitoses) were available for very few cases and therefore they will not be reported. Proliferative tumours, according to the Trouillas’ classification [72] could be identified in 89.5% of men (*n* = 17/19) and 81.8% of women (*n* = 9/11) at first available surgery, without significant difference.

Trouillas’ grade could not be assessed due to the very low number of patients for whom information on both invasion and proliferative markers was available.

Across all the studies, 21.9% (*n* = 16/73) deaths were registered which corresponded to an identical mortality rate in both sexes and time to death from diagnosis was similar in men and women (Table 1). However, outcome data were missing for 14 men and 6 women.

#### Response to APT/PC specific treatments

Temozolomide was administered as first-line non-standard treatment in 100% of men and in 91.7% of women with available data on treatment regimen (Table 1). Following disease progression on TMZ, additional therapeutic options were employed: ICIs (pembrolizumab, nivolumab and ipilimumab) and bevacizumab (BVZ) were the most common, while chemotherapy, mTOR inhibitor everolimus and PRRT were used in a minority of patients (Table 1).

**Fig. 1:**
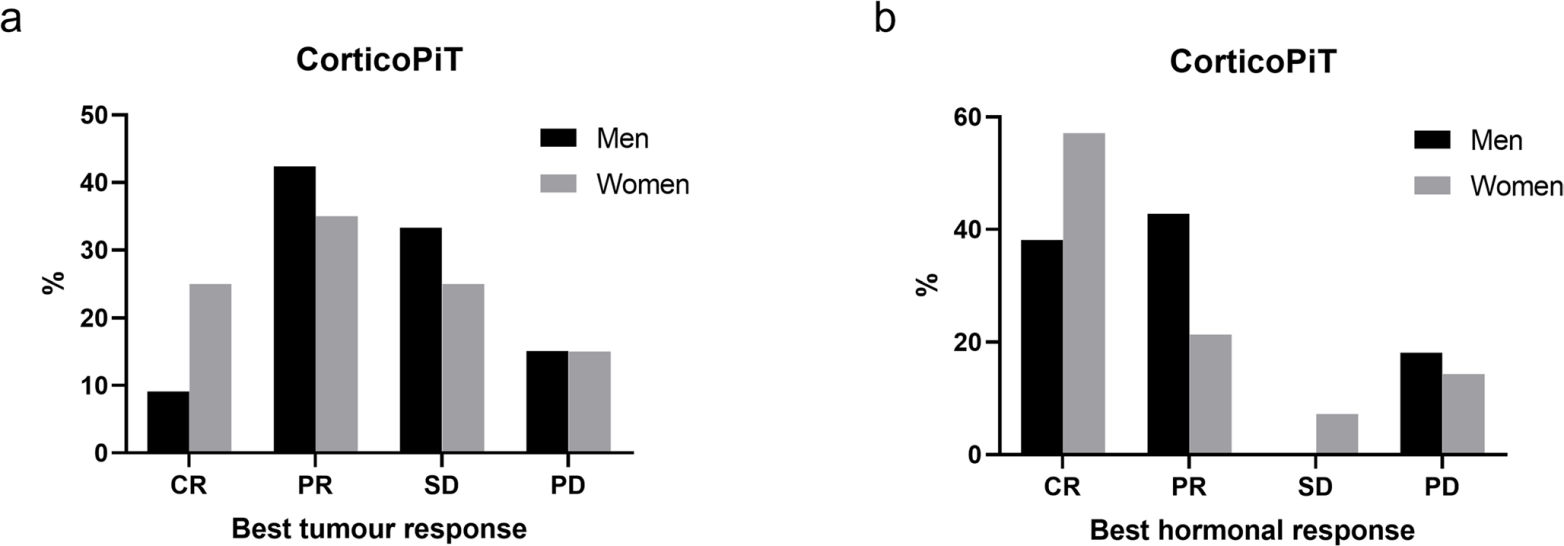
Best tumour and hormonal response to temozolomide treatment according to sex in corticoPiT. (**a**) Tumour responses. Men: CR: 9.1%, PR: 42.4%, SD: 33.3%, PD: 15.1%; women: CR: 25.0% in PR: 35.0%, SD: 25.0%, PD: 15.0%. (**b**) Hormonal responses. Men: CR 38.1%, PR: 42.8%, SD: 0%, PD: 18.1%; women: CR 57.1%, PR: 21.4%, SD: 7.2%, PD: 14.3%. Tumour and hormonal responses are established according to the RECIST criteria for solid tumours. The number of responders is expressed in percentage (%) of patients with available information. Abbreviations: CR: complete response; PR: partial response; SD: stable disease; PD: progressive disease

Tumour and hormonal responses to temozolomide treatment are shown in Fig. 1 and best tumour and hormonal responses for each patient are showed in Supplementary Table 2.

Tumour complete response (CR) was observed in 22.2% (*n* = 6/27) of women and 4 of them showed associated hormonal CR. CR was sustained in 83.3% (5/6) at last follow-up (median 27.5 months, IQR 22.5–50.5). In men, 9.1% of tumour CR (*n* = 4/44) were reported and concordant hormonal CR was seen in 75% of those with tumour CR (*n* = 3/4). These responses were mainly obtained on TMZ treatment (Fig. 1) All men tumour CR and concordant hormonal responses were sustained (Supplementary Table 2) at last follow-up (median 42.5 months, IQR 33.7–48.7).

Best tumour response was partial response (PR) in 37.0% of women (*n* = 10/27). Associated hormonal responses were: 5 CR and 3 PR. In 2 patients with initial PR, disease finally progressed; while in one with initial associated hormonal CR a mild biochemical progression was seen that led to a final PR when final hormonal values were compared with pre-treatment ones (Supplementary Table 2).

Tumour PR was described in 40.9% of men (*n* = 18/44) and concordant hormonal PR was observed in 4 cases. In 7 patients hormonal CR was recorded, instead. Metastatic disease developed or progressed in 4 men during or after the completion of treatment, despite an initial tumour PR (Supplementary Table 2). Both tumour and hormonal progression were observed after initial PR or CR in 3 patients, while only tumour progression was observed in one patient with initial PR and hormonal CR.

Of note, in some cases only general description of treatment response was available and they were excluded by the treatment response evaluation. In 2 patients (one woman and one man) tumours were reported to be “responsive” to temozolomide treatment [34]; one man was reported to be “free from tumour progression and metastases” [44]; normal cortisol levels after temozolomide were described in one woman [62].

Stable disease (SD) as best tumour response was observed in 25.9% of women (*n* = 7/27). One patient had a concordant hormonal SD, whereas 3 of them showed better hormonal than tumour response: 2 were PR and one was a CR. Among women with SD, one progressed after TMZ, and one showed a better response on metastases (PR) than on primary tumour after TMZ (Supplementary Table 2).

In men, tumour SD as best response was reported in 36.4% (*n* = 16/44). Seven hormonal responses were available, and they were all better than tumour response: 2 were CR and 5 were PR. Four patients showed tumour progression, in one case accompanied by hormonal progression, after initial response. In one patient instead, tumour remained stable despite hormonal progression. 

Hormonal SD only was observed in one further patient (man), while his tumour never responded to treatments. Hormonal SD was not sustained in this case, and the patient eventually progressed.

Focusing on hormonal responses: 19 were available in women and 27 in men. CR were 52.6% in women (*n* = 10) and 44.4% in men (*n* = 12); PR were 26.3% in women (*n* = 5) and 37.5% in men (*n* = 9), SD were one in women and one in men.

Among women, 4 patients (4/27, 14.8%) never responded to any treatment and tumour progression was associated with hormonal progression in four of them (information was not available for one); 6 tumours in men (6/44, 13.7%) always progressed despite treatments. One of these cases showed initial and not sustained hormonal SD, as discussed earlier. The remaining 5 have always shown hormonal progressive disease.

In women it may seem that there is a tendency to higher tumour CR with respect to men (22.2% vs. 9.1%), but this was not confirmed by ꭓ^2^ test. No relevant differences were found concerning hormonal responses. Final tumour and hormonal PD were observed in similar proportion of men (29.5% for tumour and 33.3% for hormones) and women (29.6% for tumour and 31.5% for hormones) and no statistical difference was found. In general tumour and hormonal responses were concordant and positive tumour responses were associated with an equal or better hormonal response.

### Lactotroph APT/PC

A total of 80 patients with lactoPiT were included, comprising of 47.5% (*n* = 38) APTs and 52.5% (*n* = 42) PCs. Among them, 63.8% were men [28, 34, 38, 42, 52, 73–100] and 36.2% were women [27, 28, 34, 42, 52, 75, 82, 89, 91, 94, 101–111] (Supplementary Table 1). Relevant clinical information is shown in Table 2.

**Table 2:** Clinical and pathological features in lactoPiT according to sex

Clinical features	Total	Men	Women	*P*
Number of cases	80	51 (63.7%)	29 (36.3%)	NA
APT, *n*	38	26 (68.4%)	12 (31.6%)	NA
PC, *n*	42	25 (59.5%)	17 (40.5%)	NA
Age at diagnosis (*n* = 79/50/29)	47 [30.0–60.0]	45.5 [28.7–60.0]	49 [30.5–59.5]	0.36
Age at metastases (*n* = 31/18/13)	57 [45.0-72.0]	56.5 [43.0–70.0]	58 [52.0–71.0]	0.63
Metastases (*n* = 42/25/17)	Bone 18 (42.8%) Lymph 5 (11.9%) Meningeal 4 (9.5%) Intracranial/brain 16 (38.1%) Other 5 (11.9%)	Bone 9 (36.0%) Intracranial/brain 11 (44.0%) Lymph 2 (8.0%) Meningeal 3 (12.0%) Other 1 (4.0%)	Bone 9 (52.9%) Intracranial/brain 5 (29.4%) Lymph 3 (17.6%) Meningeal 1(5.9%) Other 4 (23.5%)	NA
Functioning at diagnosis	71/80 (88.7%)	44/51 (86.3%)	27/29 (93.1%)	0.70
NF to F	3/80 (3.7%)	3/51 (5.9%)	0 (0%)	0.55
F to NF	1/80 (1.2%)	1/51 (1.9%)	0 (0.0%)	> 0.99
Macrotumour at diagnosis	38/53 (71.7%)	23/33 (69.7%)	15/20 (75.0%)	0.76
Invasion at diagnosis	21/28 (75.0%)	14/18 (77.8%)	7/10 (70.0%)	0.67
Invasion (after)	24/25 (96.0%)	17/18 (94.4%)	7/7 (100%)	> 0.99
First line treatment	DA 42/74 (56.8%) Surgery 25/74 (33.8%) Radiotherapy 1/74 (1.4%)	DA 27/49 (55.1%) Surgery 19/49 (38.8%) Radiotherapy 1/49 (2.0%)	DA 15/25 (60.0%) Surgery 6/25 (24.0%) Radiotherapy 0 (0.0%)	NA
Pituitary surgeries (median number)	2 [1–3]	2 [1–3]	2 [1–3]	0.84
Radiotherapy	62/75 (82.7%)	43/49 (87.8%)	19/26 (73.1%)	0.12
Baseline prolactin (ng/ml)	735 [306.8–2034.8.8.8]	1107.5 [280.0–2870.5.0.5]	585.5 [297–958]	0.29
Secondary DA resistance	27/48 (56.2%)	14/30 (46.7%)	13/18 (72.2%)	0.13
Follow-up duration (*n* = 57/37/20)	10.8 [7–21.5]	13 [8–21]	7.7 [6–15]	0.41
Deaths	19/72 (26.4%)	13/48 (27.1%)	6/24 (25.0%)	> 0.99
Time to death from diagnosis	9.3 [6–16]	8 [6–18.5]	10.6 [4.1–25]	0.61
Pathology				
Ki67 ≥ 3% (general)	55/63 (87.3%)	33/39 (84.6%)	22/24 91.7%	0.70
Ki67 ≥ 10% (general)	40/63 (63.5%)	25/39 (64.1%)	15/24 (62.5%)	> 0.99
Ki67 ≥ 3% (before)	44/59 (74.6%)	25/37 (67.6%)	19/22 (86.4%)	0.13
Ki67 ≥ 10% (before)	24/59 (40.7%)	15/37 (40.5%)	9/22 (40.9%)	> 0.99
Ki67 ≥ 3% (after)	21/24 (87.5%)	13/15 (86.7%)	8/9 (88.9%)	> 0.99
Ki67 ≥ 10% (after)	18/24 (75.0%)	11/15 (73.3%)	7/9 (77.8%)	> 0.99
Proliferative (general)	25/43 (58.1%)	17/29 (58.6%)	8/14 (57.1%)	> 0.99
APT/PC specific treatments				
Temozolomide	62/80 (77.5%)	41/51 80.4%	21/29 (72.4%)	0.42
ICI	16/80 (20.0%)	8/51 (15.7%)	8/29 (27.6%)	0.25
BVZ	5/80 (6.2%)	2/51 (3.9%)	3/29 (10.3%)	0.35
Everolimus	6/80 (7.5%)	4/51 (7.8%)	2/29 (6.9%)	> 0.99
PRRT	2/80 (2.5%)	1/51 (2.0%)	1/29 (3.4%)	> 0.99

Continuous variables are expressed as median with interquartile range; age, follow-up duration and time to death are expressed in years. Dichotomous and ordinal variables are expressed as frequencies and percentages. Comparisons between sexes for continuous variables were performed using the Mann-Whitney U test for unpaired data, and differences in dichotomous variables were assessed by Fisher’s exact test or ꭓ^2^ test whenever appropriated. Statistical significance was defined as a p-value < 0.05

*APT* aggressive pituitary tumour, *BVZ* bevacizumab, *DA* dopamin agonist, *F* functioning, *ICI* immune checkpoint inhibitors, *NA* not available, *NF* non-functioning, *PC* Pituitary Carcinoma, *PRRT* peptide receptor radionuclide therapy

“Before” and “after” refer, respectively, to the period before and after the evidence of aggressive/malignant behaviour

Both APTs and PCs showed male predominance, with 68.4% and 59.5% respectively. The median age at diagnosis was slightly lower in men (45.5 years, IQR 28.7–60.0) than in women (49 years, IQR 30.5–59.5). Median follow-up duration was longer in men (13 years, IQR 8–21) than in women (7.7 years, IQR 6–15), but not statistically significant. Age at metastasis in PCs was similar between sexes, with a median of 7 years (IQR 45.0–72) (Table 2). This finding differs from well-established data showing earlier diagnosis in women due to symptomatic presentation, especially in reproductive age [112, 113]. The most frequent location of metastases differed between sexes: in men, they were predominantly cerebral (44%, *n* = 11/25) whereas in women mainly skeletal (52.9%, *n* = 9/17) (Table 2).

Regarding functioning status, most patients had functioning tumours (86.3% in men and 93.1%, in women). Secretory status after aggressive transformation shifted exclusively in men: only one functioning tumour became non-functioning during temozolomide treatment [38], whereas 42.9% (*n* = 3/7) of NF became functioning later [81, 83, 96]. In contrast, women kept their functional status. Conversion of a non-functioning tumour to a functioning prolactinoma is rarely discussed in literature and is not considered as a marker of aggressiveness in current guidelines [114]; however, such a shift has been associated to aggressiveness in individual reports [3].

Tumour volume data were available in 66.3% (*n* = 53/80) of patients in our cohort, 33 men and 20 women. Most APTs and PCs were macrotumours occurring in 69.7% of men and 75.0% of women (Table 2). Microadenomas were identified in 3.0% (*n* = 1/33) of men and 20.0% (*n* = 4/20) of women, whereas giant adenomas were observed more frequently in men (27.3%, *n* = 9/33) than women (5%, *n* = 1/20). These findings align with previously published data [112].

Data on tumour invasion at diagnosis were available for 28 patients. Invasive disease was present in 77.8% of men (*n* = 14/18) and 70.0% of women (*n* = 7/10), without significant sex-related differences. After evidence of aggressive behaviour, invasion was documented in 94.4% of men (*n* = 17/18) and all of women, similarly with no significant differences between sexes (Table 2). These findings indicate that invasion was highly prevalent in both sexes and became nearly universal following the development of aggressive behaviour. In contrast, existing evidence shows that men more frequently present with invasive disease [112]. Baseline PRL levels at diagnosis were higher in men (median 1107.5 ng/ml, IQR 280–2870.5.5) than in women (median 585.5 ng/ml, IQR 297–958 ng/ml), in line with existing evidence [112, 115], but without statistical difference.

Conventional treatments for lactoPiT include DA, surgery and radiotherapy [114] (Table 2). All patients except one received standard first-line treatment. This was the case of a 17-year-old man with an invasive macroadenoma and an impaired vision and visual field who firstly received gamma knife stereotactic radiosurgery, with amelioration of his symptoms, and afterwards he received DA [97]. The proportion of secondary resistance to DA was higher in women than in men (72.2%, *n* = 18 vs. 46.7%, *n* = 30); however, this difference did not reach statistical significance.

Information on surgery was available for all patients, and all underwent pituitary surgery, with a median number of 2 procedures for both men and women (IQR 1–3). In only one reported case of a microprolactinoma [110], transsphenoidal surgery was performed as first-line treatment; however, no detailed clinical history or pathological data were available. Postoperative radiotherapy was administered in 87.7% of men and 73.1% of women (Table 2).

Pathological markers were variably reported across studies. At initial diagnosis, Ki-67 ≥ 3% was present in lower percentages of men (65.6%) than women (86%), without relevant statistical differences, while Ki-67 ≥ 10% was similar between sexes (Table 2). After evidence of aggressive behaviour, Ki-67 data from subsequent surgical specimens were available for 24 LactoPiTs (15 M and 9 W). An increase in Ki-67 to ≥ 3% was observed in *n* = 13/15 men (86.7%) and *n* = 8/9 women (88.9%). Ki-67 reached levels ≥ 10% in similar proportions of men and women during subsequent surgeries (Table 2). A decrease in Ki-67 was observed in 2 men (to 15% and 7%) and in 1 woman (to 3.5%). Baseline proliferative status after first surgery, assessed according to the Trouillas classification [72], was assessable in 29 men and 14 women, showing comparable proportions of proliferative tumours (Table 2). As per CorticoPiT Trouillas’ grade could not be assessed due to the high number of missing data. Overall, pathological data at first diagnosis are largely unavailable in microprolactinomas; notably, a markedly elevated MIB-1 index (10–15%) was reported in only one case [95], therefore no reliable proliferative predictors of unfavorable evolution can be identified.

Interestingly, one woman [42] and 2 men [42, 76] developed GH co-secretion or immunohistochemical GH positivity, despite initial clinical presentation as lactoPiT.

Reported mortality at last follow-up was similar between men and women, for a mortality rate of 26.4% on the entire cohort and the median time to death did not differ significantly between men and women (Table 2).

#### Response to APT/PC specific treatments

**Fig. 2:**
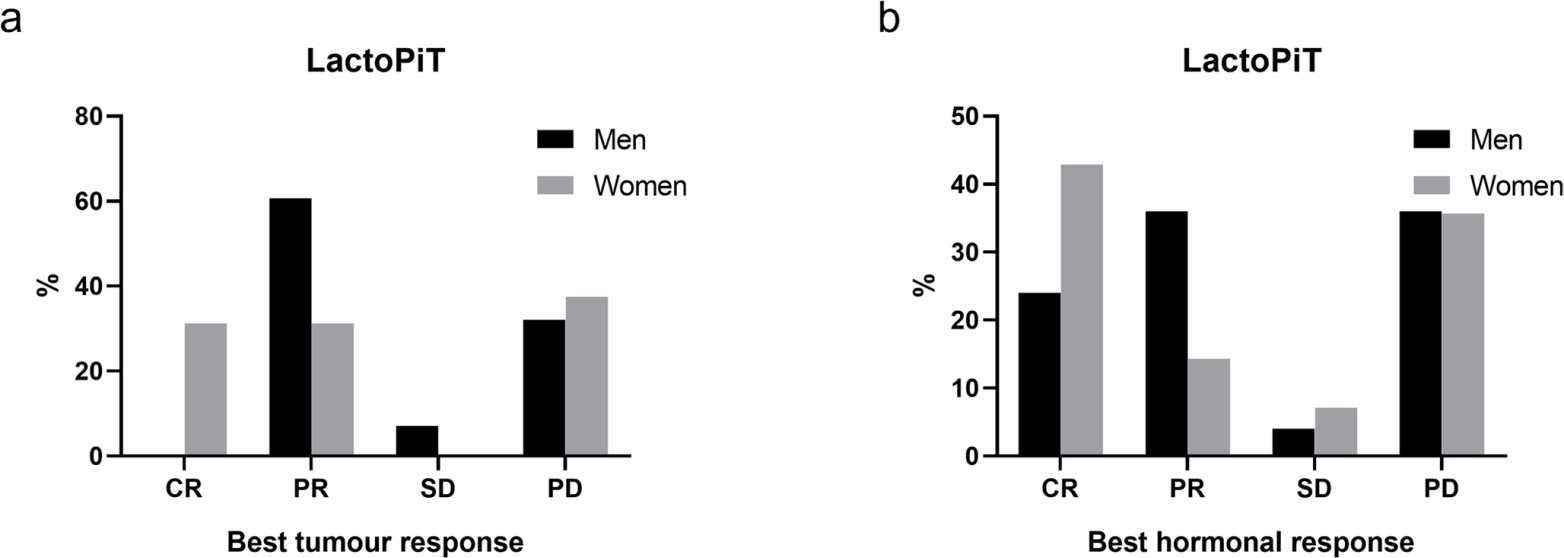
Tumour and hormonal response to temozolomide treatment according to sex in lactoPiT. (**a**) Tumour responses. Men: CR: 0%, PR: 60.7%, SD: 7.1%, PD: 32.1% women: CR: 31.2%, PR: 31.2%, SD: 0%, PD: 37.5% (**b**) Hormonal responses. Men: CR 24%, PR: 36%, SD: 4%, PD: 36%; women: CR 42.9%, PR: 14.3%, SD: 7.14%, PD: 35.7%. Tumour (**a**) and hormonal (**b**) responses are established according to the RECIST criteria for solid tumours. The number of responders is expressed in percentage (%) of patients with available information. Abbreviations: CR: complete response; PR: partial response; SD: stable disease; PD: progressive disease

As observed in corticoPiT, TMZ was the most frequently used treatment in lactoPiT, specifically in 72.4% of women (*n* = 21/29) and 80.4% (*n* = 41/51) of men. Tumour and hormonal responses after TMZ are shown in Fig. 2. Among those who did not receive TMZ, 4 out of 8 women received additional therapies (octreotide/tamoxifen combination in one [105], and lapatinib in the three others [89, 106]), while one out of ten men received lapatinib [89]. The remaining 4 women and 9 men received no further treatment. (Table 2)

After TMZ failure, the most frequent additional treatments in women were ICIs (ipilimumab, nivolumab, pembrolizumab) and carboplatin. In men, ICIs were the most common alternative treatments as well, used in 35% (*n* = 7/20) of patients with available data. Best tumour and hormonal responses and their association with final outcomes are shown in Supplementary Table 3.

Tumour CR occurred exclusively in women (22.7%, *n* = 5/22) and achieved after TMZ [42, 52, 91, 108] (Fig. 2). Among these 5 patients,3 achieved concordant hormonal CR, whereas in 2 hormonal response was not available (Supplementary Table 3). Hormonal CR in men (20.0%, *n* = 7/35) occurred in the absence of tumour CR and was observed in patients with tumour PR or SD (Supplementary Table 3) demonstrating that complete prolactin normalization was not necessarily associated with complete tumour disappearance. All reported male patients who achieved hormonal CR remained in sustained remission at last follow-up, with a median follow-up of 174 months (IQR 144–255.5.5). Among women, 6 out of 8 cases maintained CR at last follow-up for a median follow-up of 166 months (IQR 84–180 months).

PR was the most frequent tumour response in both women (36.4%, *n* = 8/22) and men (48.6%, *n* = 18/37). In women, tumour PR was associated with hormonal CR and PR, demonstrating still good radiological–biochemical concordance (Supplementary Table 3). In men, tumour PR was more frequently accompanied by hormonal PR and, in several cases, by hormonal CR, suggesting a higher degree of radiological–biochemical concordance (Supplementary Table 3). Nevertheless, discordant patterns, including hormonal PD despite tumour PR, were observed in both sexes.

Tumour SD occurred in 13.6% of women (*n* = 3/22), sustained in 2 of them and in 27.0% (*n* = 10/37) of men, sustained in 6 of them. In men, tumour SD was most frequently observed during TMZ (Fig. 2b) or targeted/immunotherapy treatment and was commonly associated with hormonal SD or PR, with occasional hormonal PD. In women, tumour SD was variably associated with hormonal PR, SD, or PD. Overall, tumour SD showed partial correlation with biochemical response, as radiological stabilization was often accompanied by hormonal stabilization or improvement, although this relationship was not consistent across all cases.

Tumour response was not sustained in 22.7% of women (*n* = 5/22) [28, 42, 52, 91, 111] and 16.2% of men (*n* = 6/37) [42, 52, 91, 111]. Hormonal response was not sustained in 20.0% of women (*n* = 4/20) [42, 52, 91, 111] and in 20% of men (*n* = 7/35) [28, 91], in 2 of whom treatment had to be stopped due to side effects [91].

Finally, tumour PD occurred in 27.2% (*n* = 6/22) of women with evaluable results [27, 34, 42, 52, 89], and hormonal PD in 30.0% (*n* = 6/20) [28, 34, 42, 89], all of whom had never responded to treatment. Tumour PD [28, 34, 42, 74, 75, 77, 79, 89, 94, 95, 100] was seen in the rest of the evaluable men (*n* = 24.3%, *n* = 9/37), in one of them partial tumour regression and decrease in PRL was seen after proton beam radiotherapy that occurred after TMZ stop [42]. In cases of tumour PD, hormonal PD was usually concordant; however, isolated discordant responses were observed [28].

In summary, treatment responses differed between sexes. Tumour CR was achieved exclusively and significantly in women (22.7%, *n* = 5/22, p: 0.02 at ꭓ^2^), despite comparable use of TMZ in both sexes. Hormonal CR was observed in both men and women but remained significantly (p: 0.03 at ꭓ^2^) more frequent in women (30.0%, *n* = 6/20) than in men (20%, *n* = 7/35). Men more often demonstrated partial responses, which were typically durable, while women showed a broader distribution of outcomes, including both complete responses and a higher proportion of progressive disease. Overall, response patterns suggest that women may achieve deeper hormonal and tumoral remission, whereas males predominantly experience stable or partial tumour shrinkage.

## Discussion

This review highlights a sex-related difference in the epidemiology, clinical presentation, pathology and treatment response of APTs and PCs, particularly among corticotroph and lactotroph lineages. In contrast to the woman predominance observed in non-aggressive corticotroph and lactotroph tumours, aggressive behaviour and malignant transformation occur predominantly in men, accounting for approximately two-thirds of reported cases in large European studies and relevant systematic reviews [3–5]. These findings may suggest that male sex represents an independent risk factor for aggressive pituitary tumour behaviour.

In aggressive and malignant corticoPiT, male predominance contrasts sharply with the well-established female predominance in Cushing’s disease. Demographic characteristics were comparable between sexes. Secretory status at diagnosis was not significantly different, although in women tumours tended to be more frequently functioning. A shift from NF to F has been associated with tumour aggressiveness [3, 4] and was equally observed in both sexes, instead men uniquely showed loss of hormonal secretion over time [116, 117]. This feature is rarely reported in literature, and has mainly been described in women without suspicion of aggressive behaviour, typically following confirmed or presumed pituitary apoplexy [118]. In contrast, in this context of aggressiveness and malignancy, loss of secretory activity was observed exclusively in men, of whom only one displayed pituitary apoplexy [46]. Hence, we hypothesize that, in men, spontaneous cessation of secretory activity may be indicative of tumour aggressiveness rather than apoplexy-related remission [119, 120]. Another feature suggestive of aggressiveness is cavernous sinus invasion. At clinical presentation, tumours in men were significantly more invasive than in women, but with progression of disease and clear evidence of aggressiveness, this difference lost its significance, suggesting that at overt aggressive/malignant state, invasion is equally present in both sexes. Conversely, women more often displayed Crooke’s cell tumours (p: 0.006), a histological subtype consistently associated with aggressive behaviour [71]. Ki-67 proliferation indices both at first available surgery and before/after evidence of aggressive behaviour did not show relevant differences, but analysis was biased by the great number of missing cases especially when an attempt at stratification according to the timing of onset of aggressiveness was made. The systematic inclusion of Ki-67 in pathological reports, could improve prognostic evaluation and provide additional data on the sex-related risk, since high Ki-67 percentages (≥ 10%) are more frequently found in APT/PC [3, 4].

While both sexes achieved concordant tumour and hormonal responses, women seem to reach more frequently complete radiological remission, mainly after TMZ treatment (Fig. 1a). These findings are consistent with the heterogeneous and often transient efficacy of TMZ reported in corticotroph tumours and may reflect underlying sex-related molecular differences [3].

In aggressive and malignant lactoPiT, male sex predominance was even more pronounced, in line with known sex differences in prolactinoma biology [11, 15]. Men more often presented with invasive, giant tumours and higher prolactin levels, features associated with dopamine agonist resistance. Despite similar exposure to TMZ, complete tumour responses occurred exclusively and significantly in women, whereas men more frequently achieved partial but durable responses. This pattern suggests greater chemosensitivity in female tumours, potentially related to higher expression of Estrogen Receptor α and a lower prevalence of resistance-associated molecular alterations such as SF3B1 mutations [17, 19, 121]. These sex-specific differences highlight that lactoPiT in men are more likely to follow an aggressive clinical course, requiring closer monitoring and potentially combination therapies, whereas in women they may respond more favourably to standard treatments.

Across both corticotroph and lactotroph tumour subtypes, overall mortality was similar between sexes, highlighting the severity of APT/PC regardless of sex once aggressive behaviour is established. Nevertheless, the distinct clinical trajectories, pathological features, and response patterns observed, emphasize the need for systematic sex-stratified analyses in future research.

This review has several limitations. It is a narrative rather than systematic; therefore, a predefined protocol was not used, which may introduce selection bias. The limited number of reported cases and their heterogeneity in clinical features, follow-up duration, treatment strategies, and outcome reporting restrict the strength of quantitative comparisons and require cautious interpretation. Additionally, pathological data are inconsistently reported, if not missing. Proliferation markers on tumour samples (Ki-67, number of mitoses, p53 immunostaining), together with radiological assessment of tumour invasion, are the mainstays of the five-tiered clinicopathological classification system, proposed by Trouillas and collegues [72]. This grading system was almost never reported in the included studies, and we could not retrospectively attribute it due to the high number of missing information. Different independent researches proved its prognostic value and proposed the grade 2b (invasive and proliferative tumour) as a definition of aggressive behaviour [122–125]. The use of this classification has been included in the most recent European recommendations to guide treatment and decision-making [1]. Therefore, we encourage pathologist, clinicians and investigators to adopt this system in both their clinical practice and the research field.

Despite the comprehensive nature of this review, several issues remain unresolved: variations in classification systems over time, differences in the assessment of histopathological markers of aggressiveness, and the lack of standardized reporting limit definitive conclusions, particularly regarding potential sex-specific predictors of tumour behaviour and treatment response.

More comprehensive and standardized prospective data are needed to address these gaps.

Although data remain limited due to the rarity of the disease and the recent introduction of molecular studies in this field, sex-related differences in pituitary tumour behaviour may potentially involve mechanisms such as sex chromosome alterations (e.g., X/Y chromosome gains or losses), epigenetic and methylation differences, and differential exposure to steroid hormones. These factors could potentially influence tumour aggressiveness and should be explored in future molecular and translational studies.

In summary, male sex is associated with a higher prevalence and more aggressive corticotroph and lactotroph APT/PC, whereas women may achieve more profound tumour and hormonal responses to TMZ, particularly in lactotroph tumours. Recognition of sex-specific differences may enhance risk stratification, surveillance strategies, and personalized therapeutic approaches in these rare but challenging pituitary neoplasms.

## Supplementary Information

Below is the link to the electronic supplementary material.

Supplementary Material 1 (DOCX 29 KB) 

Supplementary Material 2 (DOCX 24 KB)

Supplementary Material 3 (DOCX 23 KB)

## Data Availability

No datasets were generated or analysed during the current study.
