# Supplementary material for: Aggressive and malignant pituitary tumours: does the sex matter?
Source: Pituitary. 2026 Mar 7;29(2):48. doi: 10.1007/s11102-026-01656-y (PMC12967545; doi:10.1007/s11102-026-01656-y)
Supplement: Supplementary file 1 — Supplementary Material 1 (DOCX 29 KB) [file 11102_2026_1656_MOESM1_ESM.docx]

**Supplementary table 1. List of cases included in this article.**

| **CorticoPiT** | | | |
| --- | --- | --- | --- |
| **Case number** | **Author, year** | **Sex** | **Tumour type** |
| 1 | Kovàcs 2013 | F | PC |
| 2 | Lin 2018, 2021 | F | PC |
| 3 | Alshaikh, 2019 | F | PC |
| 4 | Alshaikh, 2019 | F | PC |
| 5 | Ortiz, 2012 | M | PC |
| 6 | O' Riordan, 2013 | F | PC |
| 7 | Kurowska, 2015 | F | APT |
| 8 | Touma,2017 | M | PC |
| 9 | Rotman, 2019 | M | PC |
| 10 | Osterhage, 2021 | M | APT |
| 11 | Osterhage, 2021 | M | APT |
| 12 | Majd, 2020 | M | PC |
| 13 | Majd, 2020 | F | PC |
| 14 | Majd, 2020 | M | PC |
| 15 | Ilie, 2022 | M | APT |
| 16 | Ilie, 2022 and Duhamel, 2020 | F | PC |
| 17 | Ilie, 2022 | F | APT |
| 18 | Ilie, 2022 | M | APT |
| 19 | Ilie, 2022 | F | APT |
| 20 | Ilie, 2022 | M | PC |
| 21 | Ilie, 2022 | F | APT |
| 22 | Ilie, 2022 | M | PC |
| 23 | Ilie, 2022 | M | PC |
| 24 | Caccese,2020 | M | APT |
| 25 | Sol, 2020 | M | PC |
| 26 | Shah, 2022 | M | APT |
| 27 | Jouanneau, 2012 | M | PC |
| 28 | Donovan, 2016 | F | PC |
| 29 | Annamalai, 2012 | M | PC |
| 30 | Joehlin Price, 2017 | F | PC |
| 31 | Raverot, 2010 | M | PC |
| 32 | Raverot, 2010 | M | APT |
| 33 | Raverot, 2010 | M | PC |
| 34 | Raverot, 2010 | F | APT |
| 35 | Mohammed 2009 | F | APT |
| 36 | Mohammed 2009 | M | PC |
| 37 | Takeshita 2009 | F | PC |
| 38 | Moyes 2009 | F | APT |
| 39 | Losa, 2010 | M | APT |
| 40 | Losa, 2010 | M | PC |
| 41 | Losa, 2010 | F | APT |
| 42 | Losa, 2010 | F | APT |
| 43 | Lamas, 2023 | M | APT |
| 44 | Lamas, 2023 | F | APT |
| 45 | Lamas, 2023 | M | APT |
| 46 | Lamas, 2023 | M | APT |
| 47 | Lizzul, 2020 | M | APT |
| 48 | Lizzul, 2020 | M | APT |
| 49 | Lizzul, 2020 | M | APT |
| 50 | Lizzul, 2020 | F | APT |
| 51 | Lizzul, 2020 | M | PC |
| 52 | Lizzul, 2020 | M | APT |
| 53 | Ceccato 2015 | M | APT |
| 54 | Ceccato 2015 | M | APT |
| 55 | Ceccato 2015 | M | APT |
| 56 | Curtò 2010 | M | PC |
| 57 | Gilis-Januszewka, 2018 | M | APT |
| 58 | Bruno, 2015 | F | APT |
| 59 | Bruno, 2015 | F | APT |
| 60 | Bode, 2010 | F | PC |
| 61 | Dillard, 2011 | M | APT |
| 62 | Bengtsson, 2015 | F | APT |
| 63 | Bengtsson, 2015 | M | PC |
| 64 | Bengtsson, 2015 | M | PC |
| 65 | Bengtsson, 2015 | M | PC |
| 66 | Stelmachowksa-Bansas | F | PC |
| 67 | Decaroli, 2021 | F | APT |
| 68 | Mendola 2014 | M | PC |
| 69 | Moshkin, 2011 | M | PC |
| 70 | Mirallas, 2021 | M | APT |
| 71 | Nakano-Tateno, 2021 | M | PC |
| 72 | Nakano-Tateno, 2021 | M | APT |
| 73 | Thearle, 2011 + Zacharia, 2014 | M | PC |
| 74 | Zacharia, 2014 | M | APT |
| 75 | Zacharia, 2014 | F | APT |
| 76 | Zacharia, 2014 | M | APT |
| 77 | Asimakopoulou, 2014 | F | APT |
| 78 | Cornell, 2013 | M | PC |
| 79 | Rotondo, 2012 | F | APT |
| 80 | Arnold, 2012 | F | PC |
| 81 | De Alcubierre, 2024 | M | PC |
| 82 | Xu, 2020 | M | PC |
| 83 | Pinchot, 2009 | F | PC |
| 84 | AbdelBaki, 2017 | F | PC |
| 85 | Hirohata, 2013 | F | Undetermined |
| 86 | Hirohata, 2013 | F | Undetermined |
| 87 | Hirohata, 2013 | M | Undetermined |
| 88 | Hirohata, 2013 | F | Undetermined |
| 89 | Burman, 2022 | M | APT |
| 90 | Burman, 2022 | M | APT |
| 91 | Burman, 2022 | F | PC |
| 92 | Burman, 2022 | F | PC |
| 93 | Burman, 2022 | M | PC |
| **LactoPiT** | | | |
| 1 | Duhamel, 2020 | M | APT |
| 2 | Lizzul, 2020 | M | APT |
| 3 | Raverot, 2012 | M | PC |
| 4 | Hirohata, 2013 | F | PC |
| 5 | Hirohata, 2013 | F | PC |
| 6 | Hirohata, 2013 | M | PC |
| 7 | Hirohata, 2013 | F | APT |
| 8 | Hirohata, 2013 | F | APT |
| 9 | Bengtsson, 2015 | M | APT |
| 10 | Bengtsson, 2015 | M | APT |
| 11 | Bengtsson, 2015 | M | APT |
| 12 | Bengtsson, 2015 | M | APT |
| 13 | Bengtsson, 2015 | M | APT |
| 14 | Bengtsson, 2015 | M | APT |
| 15 | Bengtsson, 2015 | M | APT |
| 16 | Bengtsson, 2015 | M | APT |
| 17 | Bengtsson, 2015 | F | PC |
| 18 | Bengtsson, 2015 | F | PC |
| 19 | Bengtsson, 2015 | F | PC |
| 20 | Philippon, 2012 | M | PC |
| 21 | Chen, 2017 | M | APT |
| 22 | Bettencourt-Silva, 2018 | F | PC |
| 23 | Moscote-Salazar, 2018 | F | PC |
| 24 | Seltzer, 2016 | M | PC |
| 25 | Park, 2014 | M | PC |
| 26 | Majd, 2020 | F | PC |
| 27 | Sinclair, 2019 | F | PC |
| 28 | Hong, 2020 | F | PC |
| 29 | Zemmoura, 2013 | M | PC |
| 30 | Zemmoura, 2014 | M | APT |
| 31 | Phillips, 2012 | M | PC |
| 32 | Kumar, 2006 | M | PC |
| 33 | Negron-Soto, 2004 | M | PC |
| 34 | Lim, 2006 | M | PC |
| 35 | Crusius, 2005 | M | PC |
| 36 | Byrne, 2009 | M | PC |
| 37 | Lamb, 2020 | F | PC |
| 38 | Choi, 2007 | F | PC |
| 39 | Tuleasca, 2017 | M | PC |
| 40 | Murakami, 2011 | F | PC |
| 41 | Hagen, 2009 | F | PC |
| 42 | Hagen, 2009 | M | APT |
| 43 | Syro, 2006 | M | APT |
| 44 | Neff, 2007 | F | APT |
| 45 | McCormack, 2009 | M | PC |
| 46 | Raverot, 2010 | M | PC |
| 47 | Raverot, 2010 | M | APT |
| 48 | Raverot, 2010 | M | PC |
| 49 | Raverot, 2010 | F | PC |
| 50 | Fadul, 2006 | M | PC |
| 51 | Vaquero, 2003 | M | PC |
| 52 | Petrossians, 2000 | M | PC |
| 53 | Ayuk, 2005 | M | PC |
| 54 | Cooper, 2021 | F | PC |
| 55 | Cooper, 2021 | M | APT |
| 56 | Cooper, 2021 | M | APT |
| 57 | Cooper, 2021 | F | APT |
| 58 | Cooper, 2014 | F | APT |
| 59 | Cooper, 2014 | F | APT |
| 60 | Zhang, 2019 | M | APT |
| 61 | Giuffrida, 2019 | F | APT |
| 62 | Giuffrida, 2019 | M | APT |
| 63 | Ilie, 2022 | M | APT |
| 64 | Ilie, 2022 | F | APT |
| 65 | Ilie, 2022 | M | APT |
| 66 | Ilie, 2022 | M | PC |
| 67 | Ilie, 2022 | M | APT |
| 68 | Ilie, 2022 | M | PC |
| 69 | Borhan, 2022 | F | APT |
| 70 | Lin, 2023 | F | APT |
| 71 | Lin, 2023 | M | PC |
| 72 | Lin, 2023 | M | APT |
| 73 | Lin, 2023 | F | APT |
| 74 | Davoudi, 2022 | M | APT |
| 75 | Davoudi, 2022 | M | APT |
| 76 | Medina, 2022 | M | APT |
| 77 | Master,2025 | F | PC |
| 78 | Master,2025 | M | PC |
| 79 | Agarwal,2024 | M | PC |
| 80 | Tang, 2021 | F | APT |

CorticoPiT: corticotroph pituitary tumour; LactoPiT: lactotroph pituitary tumour; M: male; F: female; APT: aggressive pituitary tumour, PC: pituitary carcinoma
