# Supplementary material for: Aggressive and malignant pituitary tumours: does the sex matter?
Source: Pituitary. 2026 Mar 7;29(2):48. doi: 10.1007/s11102-026-01656-y (PMC12967545; doi:10.1007/s11102-026-01656-y)
Supplement: Supplementary file 2 — Supplementary Material 2 (DOCX 24 KB) [file 11102_2026_1656_MOESM2_ESM.docx]

**Supplementary Table 2**. Treatment responses in CorticoPiT

| Men | | | | | | | |
| --- | --- | --- | --- | --- | --- | --- | --- |
| Case (author, year) | Treatment | Best response | | Final response | | | Follow up from the start of the treatment of interest (months) |
|  |  | T | H | T | | H |  |
| Zacharia, 2014 | CAPTEM | CR | / | = | | | 45 |
| Shah, 2022 | ICI | CR | CR | = | | | 15 |
| Losa, 2010 | TMZ | CR | CR | = | | | 40 |
| Touma, 2017 | TMZ+BVZ+RT, TMZ | CR | CR | = | | | 60 |
| Ilie, 2022 | ICI | PR | CR | = | | | 7 |
| Cornell, 2013 | CHT | PR | CR | MTX | | = | 5 |
| Majd, 2020 | PEMBRO | PR | CR | = | | | 42 |
| Lizzul, 2020 | TMZ | PR | CR | PD | | PD | 36 |
| Thearle, 2011 and Zacharia, 2014 | TMZ | PR | CR | MTX | | = | 5.5 |
| Ceccato, 2015 | TMZ | PR | CR | = | | | 12 |
| Dillard, 2011 | TMZ | PR | CR | SD | | = | 12 |
| Mirallas, 2021 | CAPTEM> TMZ | PR | PR | = | | | 18 |
| Mohammed, 2009 | TMZ | PR | PR | MTX | | = | 21 |
| Bengtsson, 2015 | TMZ | PR | PR | PD | | PD | 8 |
| Gilis-Januszewka, 2018 | TMZ+ Keto | PR | PR | PD | | PD | 11 |
| Nakano-Tateno, 2021 | CAPTEM | PR | / | = | | | 27 |
| Nakano-Tateno, 2021 | CAPTEM | PR | / | = | | | 34 |
| Ilie, 2022 | ICI | PR | / | MTX | | / | 5 |
| Ceccato, 2015 | TMZ | PR | / | = | | | Not available |
| Curtò, 2010 | TMZ | PR | / | = | | | 17 |
| Lizzul, 2020 | TMZ | PR | / | = | | | 24 |
| Bengtsson, 2015 | TMZ | PR | / | = | | | Not available |
| Ceccato, 2015 | TMZ+PASI | SD | CR | = | | | Not available |
| Caccese, 2020 | TMZ+PASI | SD | CR | PD | | PD | 36 |
| Zacharia, 2014 | CAPTEM | SD | PR | = | | PD | 54 |
| Sol, 2020 | ICI+ Keto | SD | PR | = | | | 12 |
| Annamalai, 2012 | TMZ | SD | PR | = | | | 15 |
| Lizzul, 2020 | TMZ | SD | PR | = | | | 51 |
| Lizzul, 2020 | TMZ | SD | PR | = | | | 16 |
| Losa, 2010 | Etoposide | SD | / | = | | | 12 |
| De Alcubierre, 2024 | BVZ | SD | / | = | | | 8 |
| Ortiz, 2012 | BVZ | SD | / | = | | | 26 |
| Majd, 2020 | PEMBRO | SD | / | = | | | 4 |
| Lizzul, 2020 | TMZ | SD | / | = | | | 16 |
| Xu, 2020 | TMZ | SD | / | = | | | 12 |
| Osterhage, 2021 | TMZ | SD | / | PD | | / | 19 |
| Hirohata, 2013 | TMZ | SD | / | = | | | 8 |
| Rotman, 2019 | TMZ+/-BVZ | SD | / | = | | | 96 |
| Osterhage, 2021 | BVZ | PD | SD | = | PD | | 1 |
| Jouanneau, 2012 | TMZ, EVE | PD | PD | = | | | Not evaluable |
| Mendola, 2014 | TMZ | PD | PD | = | | | Not evaluable |
| Bengtsson, 2015 | TMZ | PD | / | = | | | Not evaluable |
| Raverot, 2010 | TMZ | PD | PD | = | | | Not evaluable |
| Raverot, 2010 | TMZ | PD | PD | = | | | Not evaluable |
| Women | | | | | | | |
| Zacharia, 2014 | CAPTEM | CR | CR | = | | | 22 |
| AbdelBaki, 2017 | CHT + adrenal block and replace | CR | CR | MTX* | =* | | 108 |
| Takeshita, 2009 | TMZ | CR | CR | = | | | 24 |
| Bruno, 2015 | TMZ+/-Keto | CR | CR | = | | | 57 |
| Bruno, 2015 | TMZ | CR | / | = | | | 31 |
| Hirohata, 2013 | TMZ | CR | / | = | | | 20 |
| Lin 2018, 2021 | IPI+NIVO, PRRT, NIVO | PR | CR | = | | PR | 42 |
| Majd, 2020 | PEMBRO | PR | CR | = | | | 12 |
| Asimakopoulou, 2014 | TMZ+ Keto+Mety | PR | CR | = | | | 28 |
| Decaroli, 2021 | TMZ | PR | CR | = | | | 53 |
| Mohammed, 2009 | TMZ | PR | CR | = | | | 16 |
| Ilie,2022 and Duhamel, 2020 | ICI | PR | PR | PD | | PD | 14 |
| Moyes, 2009 | TMZ | PR | PR | = | | | 6 |
| Kurowska, 2015 | TMZ | PR | PR | = | | | 15 |
| Hirohata, 2013 | TMZ | PR | / | PD | | / | Not available |
| Hirohata, 2013 | TMZ | PR | / | PD | | / | Not available |
| Stelmachowska-Banaś 2022 | TMZ | SD | CR | = | | | 32 |
| O’Riordan, 2013 | BVZ+PASI | SD | PR | = | | | Not available |
| Bode, 2010 | TMZ+PASI | SD | PR | = | | | 22 |
| Losa, 2010 | TMZ | SD | SD | PD | | PD | 18 |
| Joehlin Price, 2017 | CAPTEM | SD | / | = | | | 16 |
| Ilie, 2022 | ICI | SD | / | = | | | 12 |
| Lizzul, 2020 | TMZ | SD | / | = | | | 36 |
| Losa, 2010 | TMZ | PD | PD | = | | | Not evaluable |
| Donovan, 2016 | TMZ, EVE+/-RT | PD | PD | = | | | Not evaluable |
| Kovàcs, 2013 | PRRT | PD | PD | = | | | Not evaluable |
| Rotondo, 2012 | TMZ | PD | / | = | | | Not evaluable |

Treatment responses are shown as best and final responses The column “treatment” refers to the treatment responsible of the best response.
*patient received RT and BADX during her FU and now is free of disease

*Abbreviations*: T: tumour; H: hormonal; =: final response same as best response; /: value was not available or not assessed; MTX: metastases appeared during treatment; TMZ: temozolomide, ICI: immune checkpoint inhibitors; BVZ: bevacizumab; PASI: pasireotide, KETO; ketoconazole, IPI: ipilimimumab, NIVO: nivolumab, PEMBRO: pembrolizumab, METY: metyrapone, CHT: chemotherapy, RT: radiotherapy. CR: complete response, PR: partial response, SD: stable disease, PD: progressive disease.
