# Supplementary material for: Aggressive and malignant pituitary tumours: does the sex matter?
Source: Pituitary. 2026 Mar 7;29(2):48. doi: 10.1007/s11102-026-01656-y (PMC12967545; doi:10.1007/s11102-026-01656-y)
Supplement: Supplementary file 3 — Supplementary Material 3 (DOCX 23 KB) [file 11102_2026_1656_MOESM3_ESM.docx]

**Supplementary Table 3.** Treatment responses in LactoPiT

| Men | | | | | | |
| --- | --- | --- | --- | --- | --- | --- |
| Case (author, year) | Treatment | Best response | | Final response | | Follow up from the start of the treatment of  interest (months) |
|  |  | T | H | T | H |  |
| Duhamel, 2020 | BVZ | SD | SD | = | | 2 |
| Lizzul, 2020 | TMZ | SD | SD | PD | PD | 14 |
| Raverot, 2012 | TMZ | PR | PR | = | | Not available |
| Hirohata, 2013 | ΤΜΖ | PR | / | = | | Non evaluable |
| Bengtsson, 2015 | TMZ | PR | / | = | | Not available |
| Bengtsson, 2015 | ΤΜΖ | PR | / | PD | / | Not available |
| Bengtsson, 2015 | TMZ | SD | PR | = | | Not available |
| Bengtsson, 2015 | ΤΜΖ | PD | PD | = | | Not evaluable |
| Bengtsson, 2015 | ΤΜΖ | PR | PR | PD | PD | Not available |
| Bengtsson, 2015 | TMZ | PD | PD | = | | Not evaluable |
| Philippon, 2012 | TMZ | PR | PR | = | | 60 |
| Chen, 2017 | TMZ | PR | PR | = | | 30 |
| Zemmoura, 2013 | TMZ, CHT | PD | PD | = | | Not evaluable |
| Phillips, 2012 | TMZ | PD | PD | = | | Not evaluable |
| Lim, 2006 | TMZ | PR | CR | = | | 42 |
| Byrne, 2009 | TMZ | PR | PR | = | | 12 |
| Tuleasca, 2017 | TMZ | PR | CR | = | | 14 |
| Hagen, 2009 | TMZ | PR | CR | = | | 24 |
| Syro, 2006 | TMZ | PR | PR | = | | 10 |
| McCormack, 2009 | TMZ | PR | PR | = | | 4 |
| Raverot, 2010 | TMZ | PR | CR | = | | Not available |
| Raverot, 2010 | TMZ | PD | PD | = | | Not evaluable |
| Raverot, 2010 | TMZ | PD | PD | = | | Not evaluable |
| Fadul, 2006 | TMZ | PR | PR | = | | 25 |
| Cooper, 2021 | lapatinib | SD | PR | = | | 6 |
| Cooper, 2021 | lapatinib | SD | PD | = | | 6 |
| Zhang, 2019 | everolimus | SD | PR | = | | 12 |
| Giuffrida, 2019 | PRRT, TMZ/cyclophoshamide | PD | PD | = | | 48 |
| Ilie, 2022 | IPI/NIVO | SD | / | = | | 8 |
| Ilie, 2022 | TMZ, IPI+NIVO | PD | PD | = | | Not evaluable |
| Ilie, 2022 | NIVO | PR | CR | = | | 32 |
| Ilie, 2022 | IPI/NIVO | SD | SD | PD | SD | Not available |
| Ilie, 2022 | IPI/NIVO | SD | PR | PD | PD | Not available |
| Lin, 2023 | everolimus | SD | PR | PD | PD | 5 |
| Lin, 2023 | lapatinib/everolimus | / | PR | / | PD | 1.5 |
| Davoudi, 2022 | TMZ | PR | CR | = | | 6 |
| Davoudi, 2022 | TMZ | PR | CR | = | | 18 |
| Medina, 2022 | pazopanib | / | PR | PD | PD | 12 |
| Master,2025 | TMZ | PD | PD | = | | Not available |
| Women | | | | | | |
| Hirohata, 2013 | TMZ | CR | / | = | | Not available |
| Hirohata, 2013 | TMZ | PR | / | = | | Not available |
| Hirohata, 2013 | TMZ | CR | / | = | | Not available |
| Hirohata, 2013 | TMZ | PD | / | = | | Not evaluable |
| Bengtsson, 2015 | TMZ | CR | CR | = | | Not evaluable |
| Bengtsson, 2015 | Avastine/tronotecan, TMZ | PD | PD | = | | Not evaluable |
| Bengtsson, 2015 | TMZ | PR | PR | PD | PD | Not evaluable |
| Majd, 2020 | CHT, TMZ, PEMBRO | PD | PD | = | | Not available |
| Sinclair, 2019 | CHT, TMZ | PD | PD | = | | Not evaluable |
| Lamb, 2020 | NIVO | SD | SD | = | | 22 |
| Murakami, 2011 | TMZ | PR | CR | PD | PD | 22 |
| Hagen, 2009 | TMZ | PR | CR | = | | 57 |
| Neff, 2007 | TMZ | PR | PR | = | | 26 |
| Raverot, 2010 | TMZ | PD | PD | = | | Not evaluable |
| Cooper, 2021 | TMZ, lapatinib | PD | PD | = | | Not evaluable |
| Cooper, 2014 | lapatinib | PR | PR | = | | 6 |
| Cooper, 2014 | lapatinib | SD | PR | = | | 6 |
| Giuffrida, 2019 | PRRT | PR | PR | = | | 24 |
| Ilie, 2022 | IPI/NIVO | SD | PD | PD | = | 13 |
| Borhan, 2022 | TMZ | / | SD | = | | 9 |
| Lin, 2023 | cisplatin/etoposide | PR | PR | PD | PD | 20.5 |
| Lin, 2023 | TMZ | CR | CR | PD | PD | Not evaluable |
| Master, 2025 | TMZ | / | CR | = | | 22 |
| Tang, 2021 | TMZ | CR | CR | = | | 12 |

Treatment responses are shown as best and final responses The column “treatment” refers to the treatment responsible of the best response.

*Abbreviations*: T: tumour; H: hormonal; =: final response same as best response; /: value was not available or not assessed; TMZ: temozolomide, ICI: immune checkpoint inhibitors; BVZ: bevacizumab; IPI: ipilimimumab, NIVO: nivolumab, PEMBRO: pembrolizumab, CHT: chemotherapy.

CR: complete response, PR: partial response, SD: stable disease, PD: progressive disease
